# Radiation-induced apoptosis in microvascular endothelial cells.

**DOI:** 10.1038/bjc.1997.119

**Published:** 1997

**Authors:** R. E. Langley, E. A. Bump, S. G. Quartuccio, D. Medeiros, S. J. Braunhut

**Affiliations:** Joint Center for Radiation Therapy, Harvard Medical School and Dana-Farber Cancer Institute, Boston, MA 02115, USA.

## Abstract

**Images:**


British Journal of Cancer (1997) 75(5), 666–672
© 1997 Cancer Research Campaign

### Radiation-induced apoptosis in microvascular endothelial cells

**RE Langley[1], EA Bump[1], SG Quartuccio[1], D Medeiros[2] and SJ Braunhut[2]**

[1]Joint Center for Radiation Therapy, Harvard Medical School and Dana-Farber Cancer Institute, Boston, MA 02115; [2]Department of Biological Sciences, University of Massachusetts, Lowell, MA, USA

**Summary** The response of the microvasculature to ionizing radiation is thought to be an important factor in the overall response of both normal tissues and tumours. It has recently been reported that basic fibroblast growth factor (bFGF), a potent mitogen for endothelial cells, protects large vessel endothelial cells from radiation-induced apoptosis in vitro. Microvessel cells are phenotypically distinct from large vessel cells. We studied the apoptotic response of confluent monolayers of capillary endothelial cells (ECs) to ionizing radiation and bFGF. Apoptosis was assessed by identifying changes in nuclear morphology, recording cell detachment rates and by detecting internucleosomal DNA fragmentation. Withdrawal of bFGF alone induces apoptosis in these monolayers. The magnitude of this apoptotic response depends upon the duration of bFGF withdrawal. Irradiation (2–10 Gy) induces apoptosis in a dose-dependent manner. Radiation-induced apoptosis occurs in a discrete wave 6–10 h after irradiation, and radiation-induced apoptosis is enhanced in cultures that are simultaneously deprived of bFGF. For example, 6 h after 10 Gy, 44.3% (s.e. 6.3%) of cells in the monolayer simultaneously deprived of bFGF exhibit apoptotic morphology compared with 19.8% (s.e. 3.8%) in the presence of bFGF. These studies show that either bFGF withdrawal or ionizing radiation can induce apoptosis in confluent monolayers of capillary endothelial cells and that radiation-induced apoptosis can be modified by the presence of bFGF.

**Keywords:** apoptosis; ionizing radiation; endothelial cell; basic fibroblast growth factor

It has been suggested that the response of capillary endothelial cells (ECs) to ionizing radiation may be an important factor in the overall response of both normal tissues and tumours (Law, 1981; Jaenke et al, 1993). Early vascular changes following irradiation, within 24 h, include increased vascular permeability, endothelial cell swelling and neutrophil adhesion to the endothelium (Farjado et al, 1976). Morphological changes include the appearance of irregular projections of organelle-depleted EC cytoplasm protruding into the vascular lumen, as well as retraction or detachment of cells from the basement membrane accompanied by cytoplasmic swelling that can obstruct the lumen (Reinhold et al, 1990). Late effects, weeks to months following radiation, include microvessel collapse and disintegration, followed by scar formation, and can result in persistent vascular deficiency and fibrosis in the irradiated tissue (Reinhold et al, 1990).

Recent studies show that the cellular effects of ionizing radiation are more diverse than previously appreciated (Murray, 1996). Cellular pertubations initiated by ionizing radiation are not only confined to DNA damage. In vitro studies demonstrate that irradiated endothelial cells release growth factors (Witte et al, 1989) and express increased levels of adhesion and proinflammatory molecules (Hopewell et al 1993). Ionizing radiation has also been shown to induce the transcription of a number of genes and Hallahan et al (1995) recently identified the radiation-inducible promoter region of the early response gene *Egr-1*. The significance of these biochemical changes induced by irradiation and their relationship to the morphological changes, which occur in irradiated vessels, remains to be determined.

Apoptosis is an active and gene-directed mode of cell death, involved in embryological development (Abrams et al, 1993; Ellis and Horovitz, 1986) organ involution (Tenniswood et al, 1992) and the response of both normal and transformed cells to cytotoxic agents (Gorczyca et al, 1993; Fuks et al, 1994). It is characterized by rapid nuclear and cytoplasmic condensation and cellular disintegration into apoptotic bodies (Kerr et al, 1972). Recent work has shown that apoptosis is controlled by a complex network of positive and negative signals, which originate either from specific gene products or from the extracellular environment (Stewart, 1994). Apoptosis has recently been observed in a number of irradiated normal tissues, tumours and cell lines (Fuks et al, 1994; Stephens et al, 1991; Langley et al, 1994; Ling et al, 1994).

Fuks et al, (1994) have reported that large vessel bovine ECs, irradiated in suspension, undergo apoptosis after exposure to ionizing radiation and that basic fibroblast growth factor (bFGF), a potent mitogen for endothelial cells, inhibits this effect. They also found that bFGF protected against the lethal effects of radiation pneumonitis in C3H/HeJ mice and suggested that this could be attributed to bFGF protecting pulmonary endothelial cells from radiation-induced apoptosis. Tee and Travis (1995), however, did not find that bFGF protected against death from classical radiation pneumonitis in two different strains of mice. They also observed that, following irradiation, apoptotic cells were scattered throughout the lung and were not restricted to endothelial cells.

In tissues, which are dose limiting for radiation therapy, such as kidney and lung, capillary ECs are in abundance. These cells are phenotypically distinct from large vessel ECs. Apoptosis can be influenced by the extracellular matrix and cell–cell interactions

*Received 28 March 1996*
*Revised 11 September 1996*
*Accepted 18 September 1996*

*Correspondence to:* SJ Braunhut, Olsen 609, Department of Biological Sciences, University of Massachusetts, 1 University Avenue, Lowell, MA 01854, USA

**Figure 1** Phase microscopy illustrating the effect of bFGF deprivation and irradiation on capillary EC morphology. ECs were grown to confluency in the presence of bFGF (0.3 ng $ml^{-1}$). The medium was then replaced, with or without bFGF (3 ng $ml^{-1}$). Cultures were immediately irradiated (10 Gy) and 4 h later they were photographed. (**A**) Sham-irradiated cells, bFGF in the medium. (**B**) Sham-irradiated cells, bFGF removed from the medium. (**C**) Irradiated cells, bFGF in the medium. (**D**) Irradiated cells, bFGF removed from the medium

(Meredith et al, 1993). In vitro systems for studying radiation-induced apoptosis should, therefore, model the in vivo situation as closely as possible. We irradiated confluent monolayers of capillary ECs and studied the effects of bFGF.

### MATERIALS AND METHODS

### Cell cultures and irradiation

Microvessel ECs derived from bovine adrenals were kindly supplied by Dr Judah Folkman and Ms Catherine Butterfield (Children's Hospital, Boston, MA, USA). Cultures were examined for contaminating cell types by immunofluorescent determination of homogeneous expression of Factor VIII-related antigen. These cells are derived from a fresh isolate that can only remain viable and be passaged in culture for approximately 14 passages. They were grown to confluency on gelatinized tissue culture dishes (Costar, Cambridge, MA, USA) in Dulbecco's modified Eagle medium (DMEM) containing 10% calf serum, antibiotics and bFGF (0.3 ng $ml^{-1}$). As originally described by Folkman, capillary ECs require the addition of bFGF to the media to remain viable. Recombinant bFGF was a kind gift from Takeda Pharmaceutical Company, Osaka, Japan. Stock cultures were refed every 3 days. When the cells had been confluent for at least 24 h, the medium was changed to DMEM containing 5% calf serum, with or without bFGF (3 ng $ml^{-1}$) and immediately following this cells were irradiated. The higher concentration of bFGF ensured that cells were not deprived of bFGF during the experiments. This is a dose of bFGF found to be effective in averting radiation-induced apoptosis of large vessel ECs in vitro (Fuks et al, 1994). Irradiations were performed using a Philips RT-250 radiographic machine at a dose rate of 1.24 Gy $min^{-1}$ at room temperature. Control cultures, with or without bFGF (3 ng $ml^{-1}$), underwent sham irradiations.

### Morphological studies

Monolayers of ECs were fixed in situ by adding an equal volume of methanol to the spent medium. The medium/methanol was then removed and the cells were covered with methanol for at least 15 min at 4°C. The cells were stained with 4,6,diamidino-2-phenylindole (DAPI) (1 μg $ml^{-1}$), which binds to the A–T-rich regions of

© Cancer Research Campaign 1997

**Figure 2** The effect of irradiation and bFGF withdrawal on EC nuclear morphology. ECs were fixed with methanol and stained with DAPI and visualized using fluorescent microscopy 6 h after bFGF withdrawal and/or irradiation (10 Gy). (**A**) Sham-irradiated cells, bFGF in the medium. (**B**) Sham-irradiated cells, bFGF removed from the medium. (**C**) Irradiated cells, bFGF in the medium. (**D**) Irradiated cells, bFGF removed from the medium. Arrows indicate apoptotic nuclei

DNA, for 15 min at 37°C, washed once with methanol and cover-slipped using glycerol. DNA was visualized using a Nikon epifluorescent microscope equipped with a Nikon camera. The apoptotic cells were counted by two independent observers and at least 500 cells were counted for each condition. Cell detachment rates were determined by changing the media every 2 h and counting the detached cells electronically with a Coulter counter. Detached cells were collected by low-speed centrifugation, 800 × *g*, fixed with methanol, stained with DAPI and dispersed on glass slides coated with polylysine.

### DNA fragmentation

ECs were grown to confluency in T-150 gelatinized flasks in the presence of bFGF. The medium was then replaced, with or without bFGF, and the cultures were irradiated. Six hours after irradiation, the medium and the trypsinized cells were pooled and centrifuged at 800 × *g*. The cell pellets were washed in Dulbecco's phosphate-buffered saline, gently resuspended in lysis buffer (10 mM Tris, 3 mM EDTA, pH 7.4, containing 0.5% Triton X-100) and incubated for 20 min on ice. The lysates were centrifuged at 27 000 × *g* at 4°C for 20 min. DNA was then extracted from the supernatant with phenol–chloroform–isoamyl alcohol (25:24:1) and precipitated overnight at –20°C in absolute ethanol containing 0.3 M sodium acetate. Samples were treated with RNAase (200 µg ml$^{-1}$) for 10 min at 37°C followed by 5 min at 65°C, and nucleic acid was quantified by measuring absorbance at 260 nm. Samples (20 µg) were separated by gel electrophoresis (1.8% agarose gel, 75 V for 3 h) and visualized under ultraviolet illumination after staining with ethidium bromide. The standard was a 1-kb ladder (Gibco BRL, Grand Island, NY, USA).

### RESULTS

### Morphological studies

ECs were grown to confluence in the presence of bFGF in 35-mm dishes coated with gelatin. The medium was then replaced either with medium lacking bFGF or containing bFGF (3 ng ml$^{-1}$) and the cells were then irradiated ($T_0$). Cultures were examined every 2 h and photographed. Morphological changes were observed in sham cultures that were deprived of bFGF (Figure 1B) and in all

© Cancer Research Campaign 1997

**Figure 3** Effect of the duration of bFGF deprivation on EC apoptosis. ECs were grown to confluency in the presence of bFGF (0.3 ng ml$^{-1}$). The medium was then replaced either with medium containing bFGF (3 ng ml$^{-1}$) or with medium lacking bFGF. At various time points, the cells were fixed with methanol, stained with DAPI and the number of apoptotic nuclei in the monolayer counted. (■) bFGF present in the medium; (▨) bFGF absent from the medium. Values represent the mean of three separate experiments; error bars represent s.e.m

**Figure 5** Effect of irradiation and bFGF withdrawal on EC apoptosis. ECs were grown to confluency in the presence of bFGF (0.3 ng ml$^{-1}$). The medium was then replaced either with medium containing bFGF (3 ng ml$^{-1}$) or with medium lacking bFGF and the cells irradiated. Cells were fixed and stained with DAPI 6 h later and the number of apoptotic nuclei in the monolayer counted. (■) bFGF present in the medium, (▨) bFGF absent from the medium. Values represent the mean of three separate experiments; error bars represent s.e.m

**Figure 4** Internucleosomal DNA fragmentation in ECs deprived of bFGF and/or irradiated. DNA was extracted from the 27 000 × ***g*** supernatants and separated on a 1.8% agarose gel, 8 h after irradiation (10 Gy) and/or bFGF deprivation. A standard 1-kb DNA ladder (Gibco BRL, Grand Island, NY, USA) is included on the gel. Lane 1, sham-irradiated, bFGF in the medium; lane 2, 10 Gy, bFGF in the medium; lane 3, sham-irradiated, bFGF removed from the medium; lane 4, 10 Gy, bFGF removed from the medium cultures that had been irradiated (2–10 Gy) (10 Gy shown, Figure 1C and D). Within 4 h of bFGF withdrawal, a subpopulation of the cells rounded up and appeared to be above, but still attached to, the monolayer. These cells initially resisted detachment by gentle agitation (Figure 1). By 4 h, marked changes were seen in the irradiated cultures, which included rounding up of cells and the appearance of cell fragments that excluded trypan blue (Figure 1C and D).

### bFGF deprivation induces apoptosis

The morphological changes that occurred in the cultures deprived of bFGF and in those that had been irradiated suggested to us that the cells were undergoing apoptosis (programmed cell death). To test this hypothesis, nuclear morphology studies were conducted using ECs deprived of bFGF with or without irradiation and stained with DAPI (Figure 2). Figure 2B illustrates an example of the EC nuclear morphology 6 h after bFGF withdrawal and shows nuclear condensation and fragmentation, which is characteristic of apoptosis. The percentage of apoptotic cells in the monolayer was calculated at different time points after bFGF withdrawal and the results are shown in Figure 3. The number of apoptotic cells in the monolayer depended upon the duration of bFGF withdrawal. For example, 6 h after bFGF withdrawal, 2.26% (s.e. 0.14%) of the cells in the monolayer were apoptotic compared with 0.42% (s.e. 0.24%) in the control cultures. At 24 h, the percentage of apoptotic cells was 7.6% (s.e. 2.87%) in the bFGF-deprived cultures compared with 3.73% (s.e. 1.91%) in control cells.

Apoptosis involves rapid cellular condensation and fragmentation resulting in the disappearance of the cell. Our results suggest that apoptosis induced by bFGF withdrawal is a continuous process occurring over a number of days. This contrasts with the apoptotic response to ionizing radiation (see below), which was maximal 6–10 h after irradiation. All experiments were performed at least three times, and the results were reproducible with respect to the effects of either bFGF withdrawal or irradiation. A characteristic feature of apoptosis for some cells is internucleosomal DNA fragmentation (Wyllie, 1980). DNA fragmentation was just visible on our gels 8 h after bFGF withdrawal alone (Figure 4, lane 3). However, DNA ladders were clearly seen in cultures that had been deprived of bFGF for 48 h (data not shown).

© Cancer Research Campaign 1997

### Radiation-induced apoptosis occurs in the presence or absence of bFGF

ECs grown to confluency on gelatin-coated plates were irradiated (2–10 Gy) in the presence or absence of bFGF (3 ng $ml^{-1}$). The predominant changes in the irradiated cultures occurred between 6 and 10 h after irradiation. Six hours after irradiation, cells were fixed with methanol and stained with DAPI to study their nuclear morphology. Figure 2C and D shows nuclear morphology characteristic of apoptosis in cultures that had been irradiated (10 Gy) in the presence or absence of bFGF. The number of apoptotic cells in the irradiated monolayers were noted to appear increased, were counted and the results tabulated (Figure 5). Figure 5 indicates that, at 6 h, irradiated (10 Gy) ECs exhibit a marked increase in apoptosis in the presence (solid bar) or absence (stippled bar) of bFGF when compared with sham-treated cultures (0 Gy). A similar pattern was also seen at lower doses, for example 6 h after 4 Gy 5.55% (s.e. 0.45%) of the nuclei were apoptotic in cultures in which bFGF was in the medium compared with 20.25% (s.e. 5.25%) in cultures in which bFGF had been removed from the medium. Internucleosomal DNA fragmentation was also detected in cultures that had been irradiated both in the presence and in the absence of bFGF (Figure 4, lanes 2 and 4 respectively).

Apoptotic cells in vitro tend to detach from the monolayer; therefore, to quantitate the number of apoptotic cells after irradiation more accurately, we concurrently measured cell detachment and counted the number of apoptotic cells in the monolayer. Detached cells were collected by gently pipetting, fixed and stained with DAPI; more than 90% showed apoptotic nuclei (data not shown). The kinetics of cell detachment correlated closely with the number of apoptotic cells within the monolayer. For example, the number of apoptotic cells in the monolayer was maximal 6–8 h after irradiation and was greatest in the cultures that had been irradiated and simultaneously deprived of bFGF. Cell detachment was also highest in these cultures. The detached cells represent approximately 5% of the total cells on the dish. Figure 6A shows cell detachment counts for a set of cultures and Figure 6B shows the percentage of apoptotic cells in the monolayer for the same set of cultures at the end of the experiment, 8 h after irradiation. Also shown are the number of apoptotic cells in the monolayer of the identical set during the same experiment that were counted 6 h after irradiation.

**Figure 6** Quantification of apoptosis in ECs deprived of bFGF and irradiated. Cells detaching from the monolayer were counted electronically. Monolayers were fixed with methanol, stained with DAPI and apoptotic nuclei were counted. **(A)** Sham-irradiated, bFGF in the medium (□); 10 Gy, bFGF in the medium (■); sham-irradiated, bFGF removed from the medium (▲); 10 Gy, bFGF removed from the medium (●). **(B)**. Sham-irradiated, bFGF in the medium (▨); 10 Gy, bFGF in the medium (■); sham-irradiated, bFGF removed from the medium (▤); 10 Gy, bFGF removed from the medium (▥). Data shown represent one experiment performed with two identical sets of cultures processed in parallel. Set 1 was used to collect the detachment data and these monolayers were fixed at 8 h. Set 2 monolayers were fixed at 6 h to count apoptotic cells appearing in the monolayer. Increased numbers of apoptotic cells within the monolayer at 6 h correlate with increased numbers of detached cells at 8 h

### DISCUSSION

These studies demonstrate that cells from confluent monolayers of capillary ECs undergo apoptosis after exposure to ionizing radiation both in the presence and in the absence of bFGF. We also found that a percentage of capillary ECs from a confluent monolayer undergo apoptosis after bFGF deprivation alone. This latter finding is consistent with an earlier report from Araki et al (1990). These investigators found that bFGF deprivation induced apoptosis in cultures of human umbilical vein ECs.

Fuks et al (1994) have recently reported that bFGF protects bovine aortic endothelial cells (BAECs) from radiation-induced apoptosis in vitro. Our findings differ from those of Fuks et al (1994) in two ways. First, we found that bFGF deprivation induced apoptosis. Fuks et al (1992) had previously reported that BAECs had no loss of plating efficiency when deprived of exogenous bFGF for up to 5 days. This suggests that BAECs are resistant to apoptosis induced by growth factor withdrawal. Second, although we found that bFGF afforded some degree of protection from radiation-induced apoptosis, there was still a substantial apoptotic response occurring in the cultures containing bFGF, even though we used an identical and, in some experiments, higher concentration of bFGF.

© Cancer Research Campaign 1997

There were also three major differences in methodology between our study and those of Fuks et al (1992, 1994): the type of endothelial cells, the culture conditions of the cells and the method of irradiation. Their studies were with BAECs (large vessel ECs) and ours were with ECs derived from capillaries. Our studies were conducted using ECs grown on standard tissue culture plastic coated with gelatin, whereas Fuks et al (1992, 1994) conducted all their studies using ECs plated on a complex matrix derived from a mouse endodermal carcinoma cell line (PF-HR9). This matrix synthesized by carcinoma cells is not completely characterized but consists mainly of heparan sulphate, proteoglycans, laminin, collagen type IV and enactin. Their findings suggest that either BAECs do not undergo apoptosis after withdrawal of bFGF or that the matrix that the cells were plated on inhibits apoptosis induced by bFGF withdrawl. Recent work has suggested that the extracellular matrix and cell – cell interactions may influence apoptosis. For example, apoptosis in the prostate gland following hormone withdrawal does not solely depend on whether the cells express androgen receptors. Some receptor-positive cells in the proximal part of the gland survive. It has been suggested that this results from interactions with the underlying stromal cells in the proximal part of the gland and the influence of stromally-derived 'survival factors' (Tenniswood et al, 1993). It is possible that one of the components of the extracellular matrix produced by the endodermal carcinoma cell line used in Fuks' experiments was acting as a positive survival factor for the endothelial cells. Raff (1992) has suggested that all cells are programmed to die and will do so unless they receive appropriate external survival signals.

Although it is not unusual to irradiate cells in suspension and then seed them onto plates to see if they will form colonies, this may influence the apoptotic response and the accuracy of measurement. A recent report has shown that ECs grown in bacterial culture plates and therefore unable to attach to the tissue culture plastic spontaneously undergo apoptosis (Meredith et al, 1993). This effect is probably related to integrin-mediated signalling, as attachment to surfaces coated with the integrin B1 antibody, but not the non-integrin antibodies (HLA or VCAM-1), inhibited apoptosis in these cultures. We found that apoptotic ECs in suspension were particularly fragile. Apoptotic cell numbers decreased after centrifugation and were lower from suspension preparations compared with identical fixed monolayer cultures counted in parallel.

Our studies focused on the response of microvascular ECs. These cells are phenotypically distinct from large vessel endothelial cells. In tissues, which are dose limiting for radiation therapy, such as kidney and lung, capillary ECs are in abundance. We hypothesize that, although these cells may be relatively resistant to classical reproductive cell death (Hei et al, 1987), they undergo apoptosis in response to low doses of ionizing radiation. The reported incidence of radiation-induced apoptosis in vivo has been varied. A study by Meyn et al (1993) showed heterogeneity in the apoptotic response to irradiation among tumours of different histologies. They also demonstrated that the predominant wave of radiation-induced apoptosis occured 3–6 h after irradiation (Stephens et al, 1993). Fuks et al (1994) reported that, 8 h after an exposure to 20 Gy, an abundance of apoptotic nuclei could be detected in murine pulmonary capillary ECs in vivo. However, Tee and Travis (1995) examined tissue from murine lungs at 4, 8, 12 and 24 h after irradiation, and they found the maximum incidence of apoptotic cells to be 1% and noted that apoptotic cells were most frequently seen in the peribronchiolar and perivascular regions of the tissue, not in the endothelial cells. In both our study and the in vitro experiments of Fuks et al (1992, 1994), the predominant wave of endothelial cell apoptosis occured 6–8 h after irradiation.

It has been proposed that radiation damage to the microvasculature contributes to the development of late radiation effects, such as renal failure and pneumonitis (Jaenke et al, 1993). This view has been challenged by Withers et al (1980) who suggest that it is parenchymal damage rather than vascular damage, which determines late radiation effects. There are clearly marked vascular changes in irradiated tissues associated with fibrosis and atrophy. Whether this is the cause, or a result, of late tissue damage is not clear, as the cellular response to ionizing radiation is not fully understood. Traditionally, the term early effects of irradiation refers to changes that occur within the first few weeks or months after irradiation. Very early vascular changes within hours of irradiation are not prominent; however, apoptosis can be difficult to detect in vivo because the classical morphological features are only visible for a short period of time (minutes rather than hours), and it is probable that radiation-induced apoptosis in endothelial cells has been overlooked in the past. The finding that bFGF protected BAECs from radiation-induced apoptosis in vitro led Fuks et al (1992, 1994) to propose that radiation-induced pneumonitis could be avoided by protecting pulmonary endothelial cells from radiation-induced apoptosis using bFGF. However, Tee and Travis (1995) did not find that bFGF protected against classical radiation pneumonitis in two strains of mice. Our finding that microvascular ECs undergo radiation-induced apoptosis in the presence and absence of bFGF suggest that bFGF is unlikely to protect completely against radiation-induced apoptosis in vivo.

Two recent studies have emphasized the complexity and cell type-specific nature of radiation-induced apoptosis. Potten (1992) has shown that only a select group of cells within the mucosa of the small intestinal crypts respond to ionizing radiation by undergoing apoptosis. Other cells within the mucosa undergo apoptosis after exposure to chemotherapeutic agents and mutagens but appear resistant to radiation-induced apoptosis. Midgley et al (1995) studied p53 protein levels in mice after whole-body irradiation. Dramatic accumulation of p53 protein was apparent in splenocytes, thymocytes and osteocytes after irradiation, but no p53 protein was detected in hepatocytes. Induction of p53 in splenocytes and thymocytes, but not in osteocytes, resulted in apoptosis. These experiments demonstrate that the signals that control p53 induction and, hence, radiation-induced apoptosis are tightly controlled in a tissue-specific manner. Our experiments suggest that ECs from different vascular beds may differ in their susceptibility to apoptosis. Furthermore, that with respect to apoptosis, in vitro models used to study the effects of ionizing radiation should resemble as closely as possible the in vivo situation. The apoptotic response of endothelial cells and the underlying mechanisms will require further investigation and may provide interesting insights into the biological effects of ionizing radiation.

### REFERENCES

Abrams JM, White K, Fessler LI and Stellar H (1993) Programmed cell death during *Drosophila* embryogenesis. *Development* **117**: 29–43

Araki S, Shimada Y, Kaji K and Hayashi H (1990) Apoptosis of vascular endothelial cells by fibroblast growth factor deprivation. *Biochem Biophys Res Comm* **168**: 1194–1200

Ellis HM and Horovitz HR (1986) Genetic control of programmed cell death in the nematode *C elegans Cell* **44**: 817–829

© Cancer Research Campaign 1997

Farjado LF, Brown JM and Glastein E (1976) Glomerular and juxtaglomerular lesions in radiation nephropathy. *Radiat Res* **68**: 177–183

Folkman J and Moscona A (1978) Role of cellshape in growth control. *Nature* 272: 345–349

Fuks Z, Vlodavsky I, Andreeff M, McLoughlin M and Haimovitz-Friedman A (1992) Effects of extracellular matrix on the response of endothelial cells to radiation *in vitro*. *Eur J Cancer* **28A**: 725–731

Fuks Z, Persaud RS, Alferi A, McLoughlin M, Ehleiter D, Schwartz JL, Seddon AP, Cordon-Cardo C and Haimovitz-Friedman A (1994) Basic fibroblast growth factor protects endothelial cells against radiation-induced programmed cell death *in vitro* and *in vivo*. *Cancer Res* **54**: 2582–2590

Gorczyca W, Gong J, Ardelt B, Traganos F and Darzynkiewicz Z (1993) The cell cycle related differences in susceptibility of HL-60 cells to apoptosis induced by various antitumor agents. *Cancer Res* **53**: 3186–3192

Hallahan DE, Mauceri HJ, Seung LP, Dunphy EJ, Wayne JD, Hanna NN, Toledano A, Hellman S, Kufe DW and Weichselbaum RR (1995) Spatial and temporal control of gene therapy using ionizing radiation. *Nature Med* **1**: 786–791

Hei TK, Marchese MJ and Hall EJ (1987) Radiosensitivity and sublethal damage repair in human umbilical cord vein endothelial cells. *Int J Radiat Oncol Biol Phys* **13**: 879–884

Hopewell JW, Calvo W, Jaenke R, Reinhold HS, Robbins MEC and Whitehouse EM (1993) Microvasculature and radiation damage. *Recent Results Cancer Res* **130**: 1–16

Jaenke RS, Robbins MEC, Bywaters T, Whitehause E, Rezvani M and Hopewell JW (1993) Capillary endothelium: target site of renal radiation injury. *Lab Invest* **68**: 396–405

Kerr JFR, Wyllie AH and Currie AR (1972) Apoptosis: a basic biological phenomenon with wide ranging implications in tissue kinetics. *Br J Cancer* **26**: 239–357

Langley RE, Palayoor ST, Coleman CN and Bump EA (1994) Radiation-induced apoptosis in F9 teratocarcinoma cells. *Int J Radiat Biol* **65**: 605–610

Law MP (1981) Radiation-induced vascular injury and its relation to late effects in normal tissues. *Adv Radiat Biol* **9**: 37–73

Ling CC, Chang CH and Li WX (1994) Apoptosis induced at different dose rates: implication for the shoulder region of cell survival curves. *Radiother Oncol* **32**: 129–136

Meredith JEJ, Fazeli B and Schartz MA (1993) The extracellular matrix as a cell survival factor. *Mol Biol Cell* **4**: 953–961

Meyn RE, Stephens LC, Ang KK, Hunter NR, Milas L and Peters LJ (1993) Heterogeneity in apoptosis development among irradiated murine tumors of different histologies. *Int J Radiat Biol* **64**: 583–591

Midgley CA, Owens B, Briscoe C, Thomas DB, Lane DP and Hall PA (1995) Coupling between gamma irradiation, p53 induction and the apoptotic response depends upon cell type *in vivo*. *J Cell Sci* **108**: 1843–1848

Murray JC (1996) Inflammatory cytokines, radiation and endothelial gene products: a common role for reactive oxygen intermediates. In *Radiation Biology of the Vascular Endothelium*, Ruben D (ed), CRC Press: Boca Raton, FL. (in press).

Potten CS (1992) The significance of spontaneous and induced apoptosis in the gastrointestinal tract of mice. *Cancer Met Rev* **11**: 179–195

Raff MC (1992) Social controls on cell survival and cell death. *Nature* **356**: 397–400

Reinhold HS, Farjado LF and Hopewell JW (1990) The vascular system. *Adv Radiat Biol* **14**: 177–226

Stephens LC, Ang KK, Schultheiss TE, Milas L and Meyn RE (1991) Apoptosis in irradiated murine tumors. *Radiat Res* **127**: 308–316

Stephens LC, Hunter NR, Ang KK, Milas L and Meyn RE (1993) Development of apoptosis in irradiated murine tumors as a function of time and dose. *Radiat Res* **135**: 75–80

Stewart BW (1994) Mechanisms of apoptosis: integration of genetic, biochemical, and cellular indicators. *J Natl Cancer Inst* **86**: 1286–1295

Tee PG and Travis EL (1995) Basic fibroblast growth factor does not protect against classical radiation pneumonitis in two strains of mice. *Cancer Res* **55**: 298–302

Tenniswood M, Guenette S, Lakins J, Mooibroek M, Wong P and Welsh J (1992) Active cell death in hormone-dependent tissues. *Cancer Met Rev* **11**: 197–220

Tenniswood M, Taillefer D, Lakins J, Guenette S, Mooibroek M, Daehlin L and Welsh J (1993) Control of gene expression during apoptosis in hormone dependent tissue. In *Apoptosis 2: The Molecular Basis of Cell Death*, Tomei L D and Cope F O (eds), pp. 283–311 Cold Spring Harbor Laboratory Press: Cold Spring Harbor, NY.

Withers HR, Peters, LJ and Kogelnik HS (1980) The pathobiology of late effects of irradiation. In *Radiatiion Biology in Cancer Research*, Meyn R E and Withers HR (eds), pp. 439–448. Raven Press: New York.

Witte L, Fuks Z, Haimovitz-Friedman A, Vlodavsky I, DeWitt S, Goodman S and Eldor A (1989) Effects of irradiation on the release of growth factors from cultured bovine, porcine, and human endothelial cells. *Cancer Res*, **49**: 5066–5072

Wyllie AH (1980) Glucocorticoid-induced thymocyte apoptosis is assosciated with endogenous endonuclease activity. *Nature*, **284**: 555–556

© Cancer Research Campaign 1997


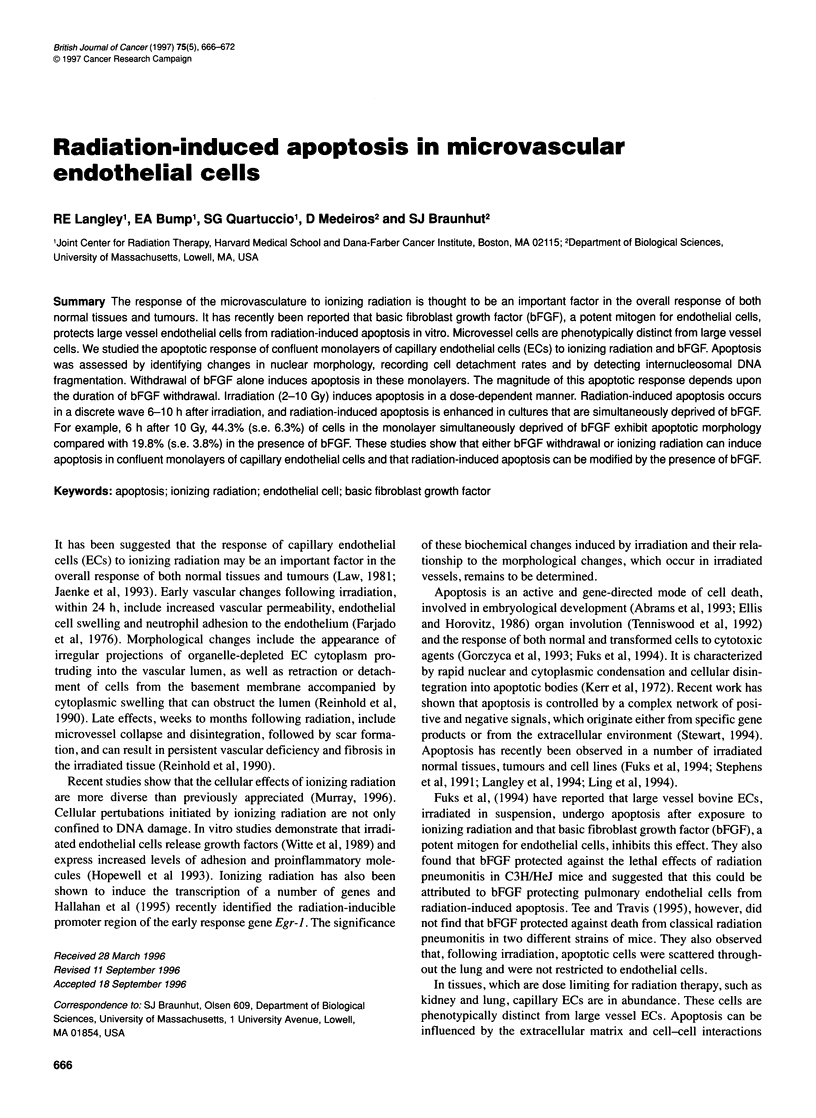

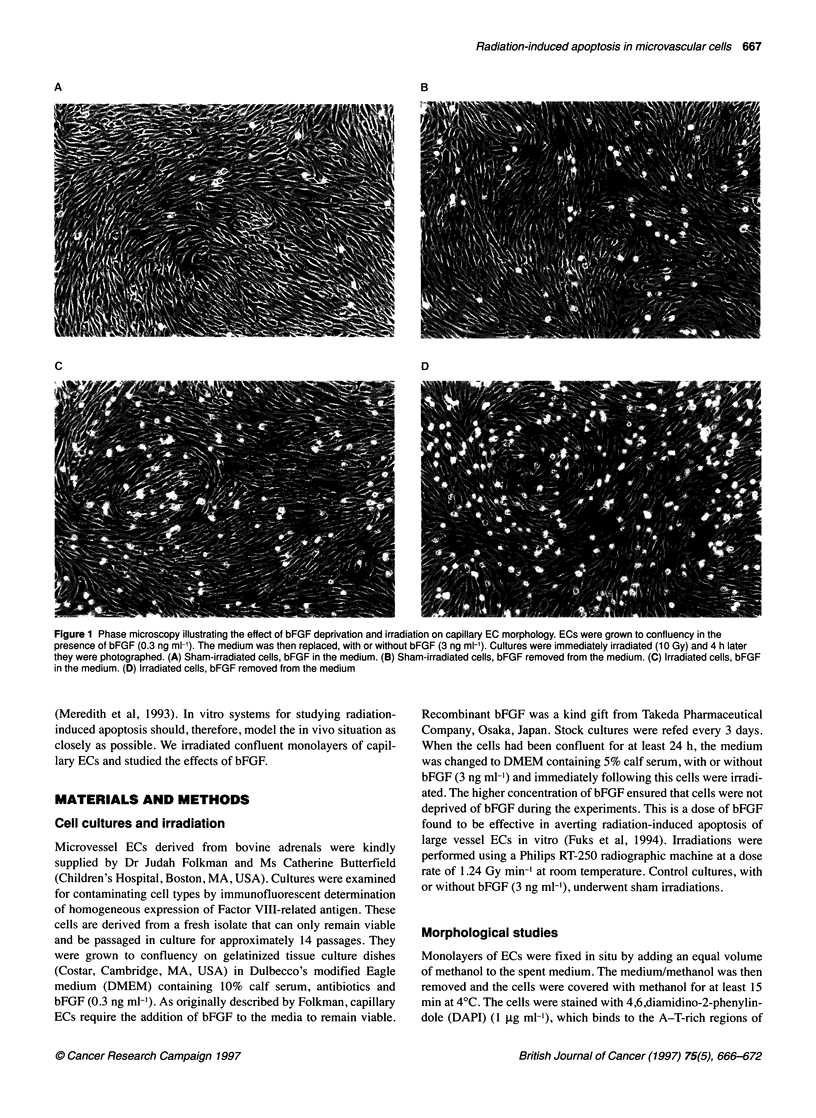

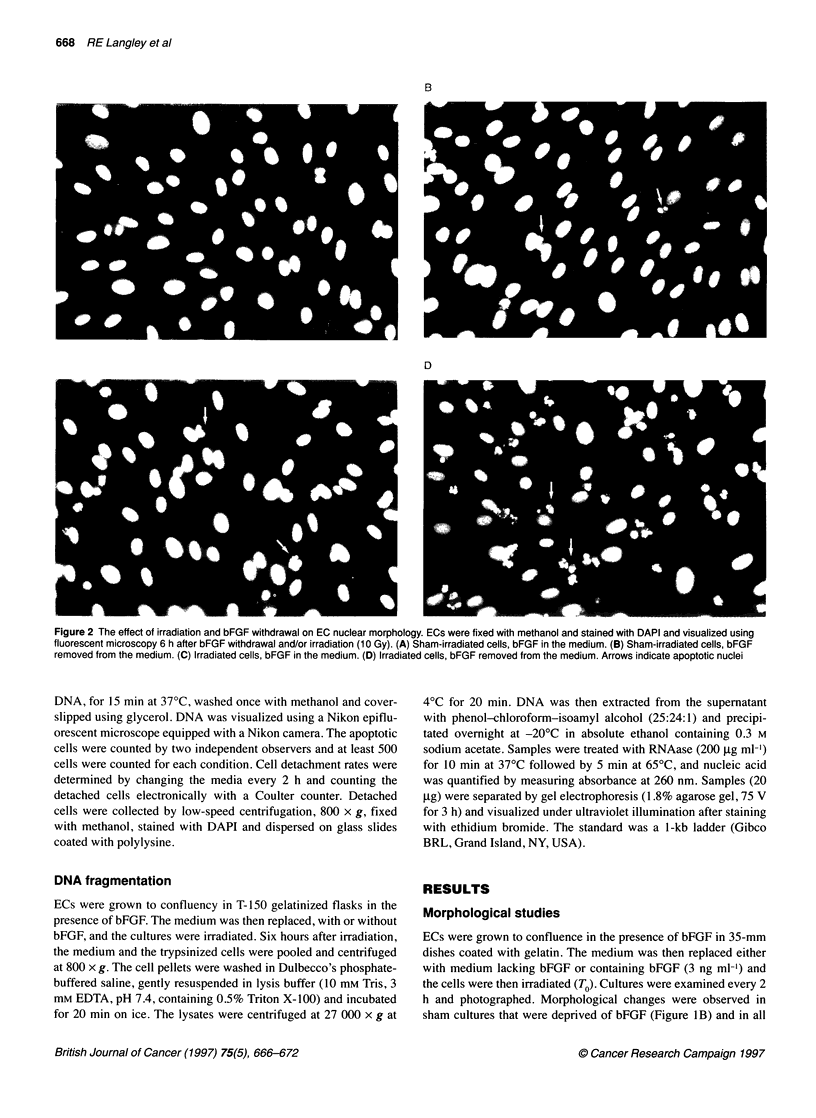

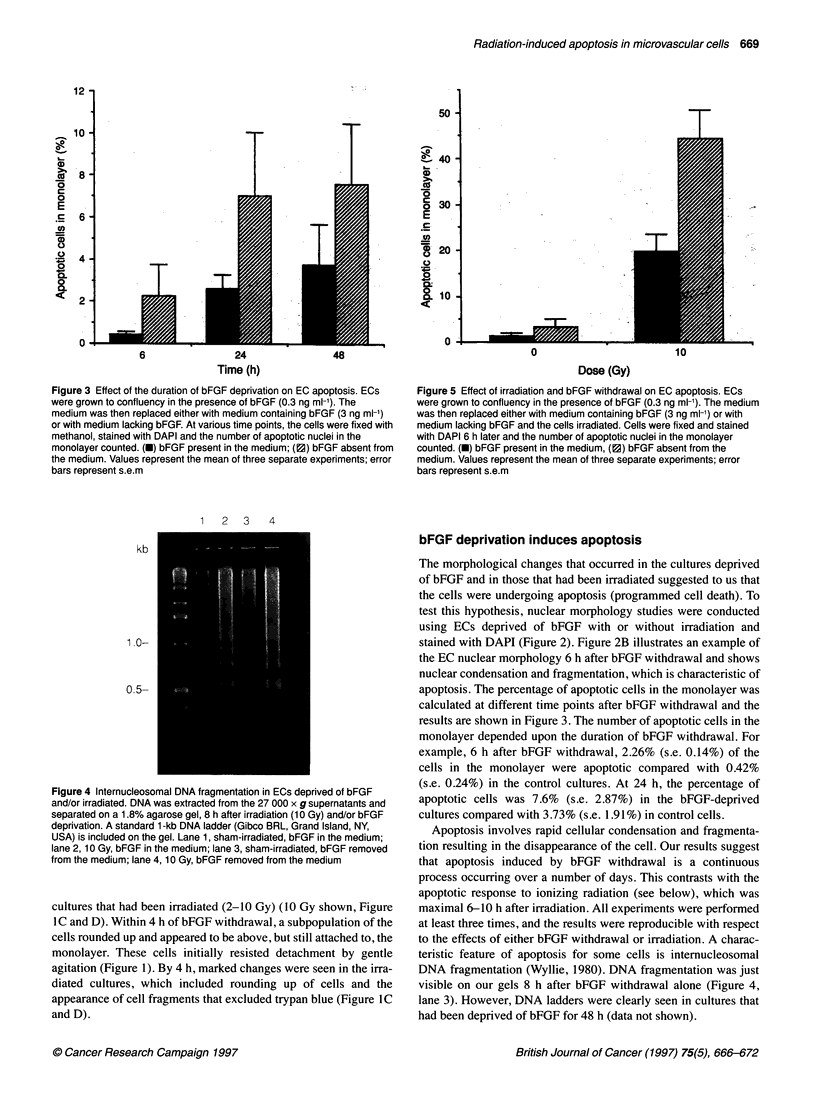

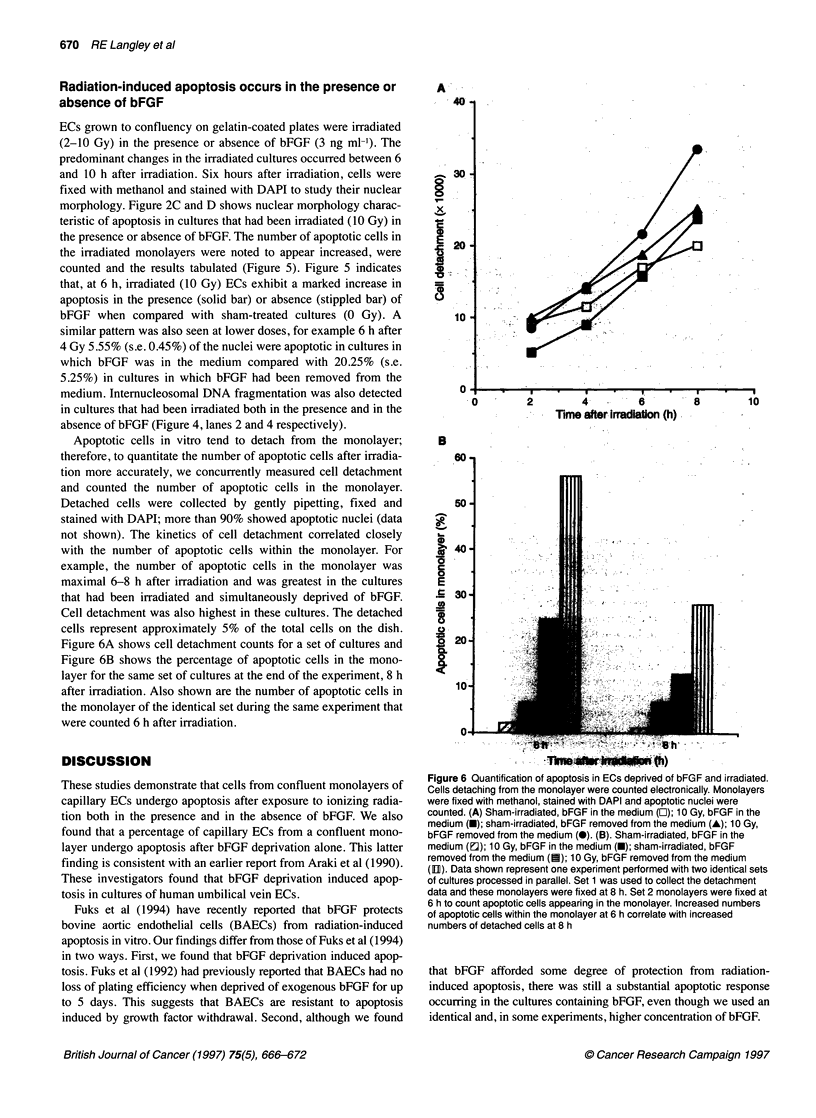

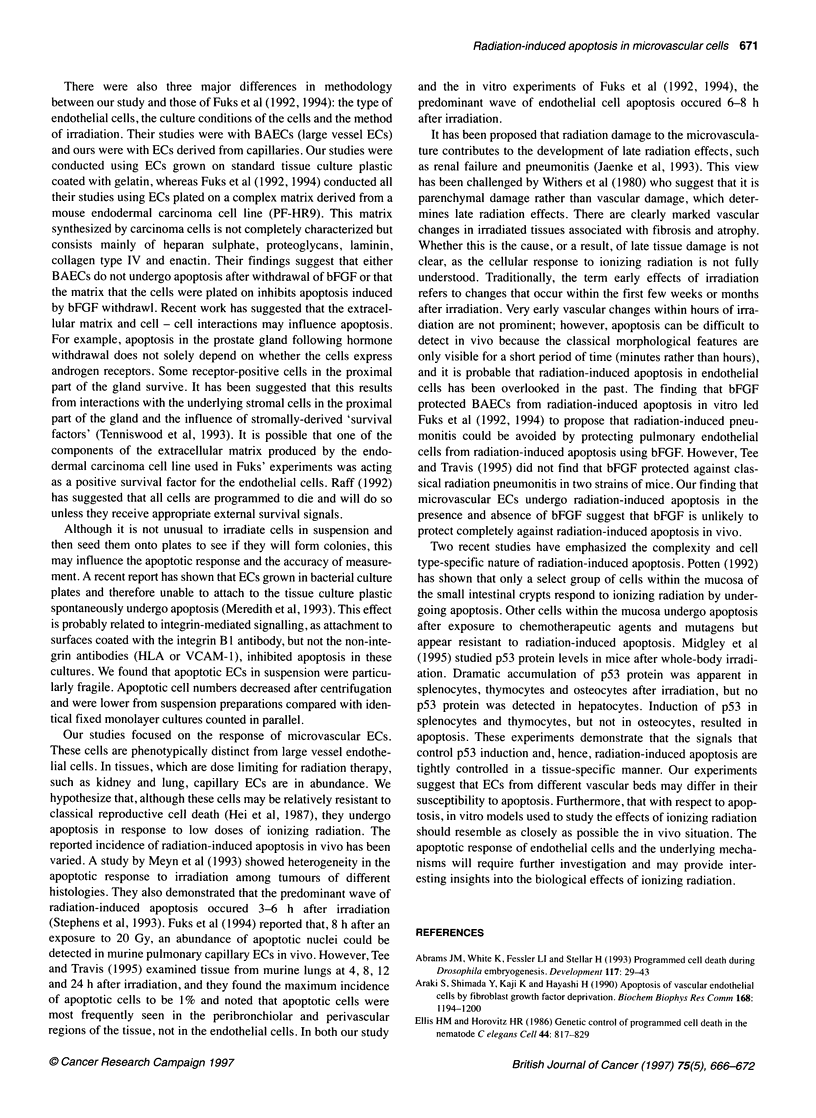

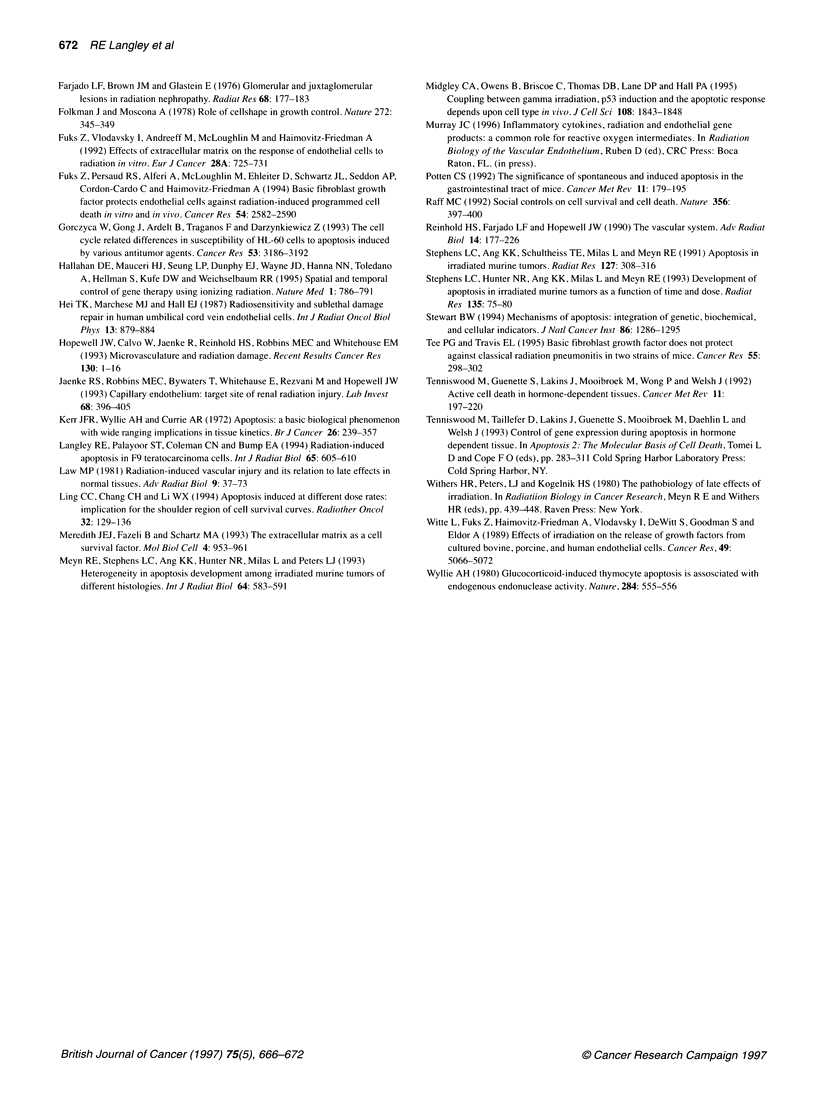


## References

[OCR_00640] Abrams J. M., White K., Fessler L. I., Steller H. (1993). Programmed cell death during Drosophila embryogenesis.. Development.

[OCR_00644] Araki S., Shimada Y., Kaji K., Hayashi H. (1990). Apoptosis of vascular endothelial cells by fibroblast growth factor deprivation.. Biochem Biophys Res Commun.

[OCR_00649] Ellis H. M., Horvitz H. R. (1986). Genetic control of programmed cell death in the nematode C. elegans.. Cell.

[OCR_00657] Fajardo L. F., Brown J. M., Glatstein E. (1976). Glomerular and juxta-glomerular lesions in radiation nephropathy.. Radiat Res.

[OCR_00661] Folkman J., Moscona A. (1978). Role of cell shape in growth control.. Nature.

[OCR_00670] Fuks Z., Persaud R. S., Alfieri A., McLoughlin M., Ehleiter D., Schwartz J. L., Seddon A. P., Cordon-Cardo C., Haimovitz-Friedman A. (1994). Basic fibroblast growth factor protects endothelial cells against radiation-induced programmed cell death in vitro and in vivo.. Cancer Res.

[OCR_00665] Fuks Z., Vlodavsky I., Andreeff M., McLoughlin M., Haimovitz-Friedman A. (1992). Effects of extracellular matrix on the response of endothelial cells to radiation in vitro.. Eur J Cancer.

[OCR_00676] Gorczyca W., Gong J., Ardelt B., Traganos F., Darzynkiewicz Z. (1993). The cell cycle related differences in susceptibility of HL-60 cells to apoptosis induced by various antitumor agents.. Cancer Res.

[OCR_00681] Hallahan D. E., Mauceri H. J., Seung L. P., Dunphy E. J., Wayne J. D., Hanna N. N., Toledano A., Hellman S., Kufe D. W., Weichselbaum R. R. (1995). Spatial and temporal control of gene therapy using ionizing radiation.. Nat Med.

[OCR_00686] Hei T. K., Marchese M. J., Hall E. J. (1987). Radiosensitivity and sublethal damage repair in human umbilical cord vein endothelial cells.. Int J Radiat Oncol Biol Phys.

[OCR_00691] Hopewell J. W., Calvo W., Jaenke R., Reinhold H. S., Robbins M. E., Whitehouse E. M. (1993). Microvasculature and radiation damage.. Recent Results Cancer Res.

[OCR_00696] Jaenke R. S., Robbins M. E., Bywaters T., Whitehouse E., Rezvani M., Hopewell J. W. (1993). Capillary endothelium. Target site of renal radiation injury.. Lab Invest.

[OCR_00701] Kerr J. F., Wyllie A. H., Currie A. R. (1972). Apoptosis: a basic biological phenomenon with wide-ranging implications in tissue kinetics.. Br J Cancer.

[OCR_00705] Langley R. E., Palayoor S. T., Coleman C. N., Bump E. A. (1994). Radiation-induced apoptosis in F9 teratocarcinoma cells.. Int J Radiat Biol.

[OCR_00713] Ling C. C., Chen C. H., Li W. X. (1994). Apoptosis induced at different dose rates: implication for the shoulder region of cell survival curves.. Radiother Oncol.

[OCR_00718] Meredith J. E., Fazeli B., Schwartz M. A. (1993). The extracellular matrix as a cell survival factor.. Mol Biol Cell.

[OCR_00722] Meyn R. E., Stephens L. C., Ang K. K., Hunter N. R., Brock W. A., Milas L., Peters L. J. (1993). Heterogeneity in the development of apoptosis in irradiated murine tumours of different histologies.. Int J Radiat Biol.

[OCR_00727] Midgley C. A., Owens B., Briscoe C. V., Thomas D. B., Lane D. P., Hall P. A. (1995). Coupling between gamma irradiation, p53 induction and the apoptotic response depends upon cell type in vivo.. J Cell Sci.

[OCR_00738] Potten C. S. (1992). The significance of spontaneous and induced apoptosis in the gastrointestinal tract of mice.. Cancer Metastasis Rev.

[OCR_00742] Raff M. C. (1992). Social controls on cell survival and cell death.. Nature.

[OCR_00750] Stephens L. C., Ang K. K., Schultheiss T. E., Milas L., Meyn R. E. (1991). Apoptosis in irradiated murine tumors.. Radiat Res.

[OCR_00754] Stephens L. C., Hunter N. R., Ang K. K., Milas L., Meyn R. E. (1993). Development of apoptosis in irradiated murine tumors as a function of time and dose.. Radiat Res.

[OCR_00759] Stewart B. W. (1994). Mechanisms of apoptosis: integration of genetic, biochemical, and cellular indicators.. J Natl Cancer Inst.

[OCR_00763] Tee P. G., Travis E. L. (1995). Basic fibroblast growth factor does not protect against classical radiation pneumonitis in two strains of mice.. Cancer Res.

[OCR_00768] Tenniswood M. P., Guenette R. S., Lakins J., Mooibroek M., Wong P., Welsh J. E. (1992). Active cell death in hormone-dependent tissues.. Cancer Metastasis Rev.

[OCR_00786] Witte L., Fuks Z., Haimovitz-Friedman A., Vlodavsky I., Goodman D. S., Eldor A. (1989). Effects of irradiation on the release of growth factors from cultured bovine, porcine, and human endothelial cells.. Cancer Res.

[OCR_00792] Wyllie A. H. (1980). Glucocorticoid-induced thymocyte apoptosis is associated with endogenous endonuclease activation.. Nature.

